# Importance of case age in the purported association between phylogenetics and hemolytic uremic syndrome in *Escherichia coli* O157:H7 infections – ERRATUM

**DOI:** 10.1017/S095026881900178X

**Published:** 2019-10-21

**Authors:** G. A. M. Tarr, S. Shringi, H. N. Oltean, J. Mayer, P. Rabinowitz, J. Wakefield, P. I. Tarr, T. E. Besser, A. I. Phipps

**Original Text**

“Similarly, the incidence of HUS in our study was 6.79 per 100 000 < 10-year-olds, compared with 0.29 per 100 000 ⩾10-year-olds,”

**Correction**

“Similarly, the incidence of HUS in our study was 0.68 per 100 000 person-years in cases <10 years, compared with 0.029 per 100 000 person-years in cases ⩾10 years.”

**Original Text**

“Individuals ⩾60-years-old have a slightly higher incidence of HUS (0.58 per 100 000) than 10–59-year-olds (0.26 per 100 000), and E. coli O157:H7 outbreaks have occurred in nursing homes [24], making this age group of particular interest,”

**Correction**

“Individuals ⩾60-years-old have a slightly higher incidence of HUS (0.058 per 100 000 person-years) than 10–59-year-olds (0.026 per 100 000 person-years), and E. coli O157:H7 outbreaks have occurred in nursing homes [24], making this age group of particular interest.”
